# Reduced Expression of Metallothionein-I/II in Renal Proximal Tubules Is Associated with Advanced Chronic Kidney Disease

**DOI:** 10.3390/toxins13080568

**Published:** 2021-08-15

**Authors:** Yi-Jhu Lu, Ya-Ju Wu, Lu-Jen Chen, Bor-Sheng Ko, Tzu-Ching Chang, Yi-Ju Wu, Shu-Man Liang, Yee-Jee Jan, Jun-Yang Liou

**Affiliations:** 1 Institute of Cellular and System Medicine, National Health Research Institutes, Zhunan 350, Taiwan; yijhu@nhri.edu.tw (Y.-J.L.); ctching9@nhri.edu.tw (T.-C.C.); Kathywu614@gmail.com (Y.-J.W.); shu-man@nhri.edu.tw (S.-M.L.); 2 Department of Pathology, Chi Mei Medical Center, Liouying, Tainan 736, Taiwan; werepast@gmail.com; 3 Department of Pathology and Laboratory Medicine, Taichung Veterans General Hospital, Taichung 407, Taiwan; lujen313@gmail.com (L.C.-J.); yejan@vghtc.gov.tw (Y.-J.J.); 4 Department of Hematological Oncology, National Taiwan University Cancer Center, Taipei 106, Taiwan; kevinkomd@gmail.com; 5 Department of Internal Medicine, National Taiwan University Hospital, Taipei 100, Taiwan; 6 Graduate Institute of Biomedical Sciences, China Medical University, Taichung 404, Taiwan

**Keywords:** ammonium pyrrolidinedithiocarbamate, aristolochic acid, chronic kidney disease, metallothionein-I/II, nephropathy

## Abstract

Chronic kidney disease (CKD) is a commonly occurring complex renal syndrome that causes overall mortality in many diseases. The clinical manifestations of CKD include renal tubulointerstitial fibrosis and loss of renal function. Metallothionein-I/II (MT-I/II) is potentially expressed in the liver and kidney, and possesses antioxidant and metal detoxification properties. However, whether MT-I/II expression is associated with the prognosis of nephropathy remains unknown. In this study, we investigated the MT-I/II level in human CKD, using immunohistochemistry. MT-I/II is located on the proximal tubules and is notably reduced in patients with CKD. MT-I/II expression was significantly correlated with the functional and histological grades of CKD. In an aristolochic acid (AAI)-induced nephropathy mouse model, MT-I/II was abundantly increased after AAI injection for 7 days, but decreased subsequently compared to that induced in the acute phase when injected with AAI for 28 days. Furthermore, we found that ammonium pyrrolidinedithiocarbamate (PDTC) restored AAI-induced MT-I/II reduction in HK2 cells. The injection of PDTC ameliorated AAI-induced renal tubulointerstitial fibrosis and reduced the concentrations of blood urea nitrogen and creatinine in mouse sera. Taken together, our results indicate that MT-I/II reduction is associated with advanced CKD, and the retention of renal MT-I/II is a potential therapeutic strategy for CKD.

## 1. Introduction

Chronic kidney disease (CKD) is characterized by the gradual loss of kidney function. It displays a decrease in the glomerular filtration rate (GFR) and an increase in urine protein excretion [1,2]. The causes of CKD include hypoxia, hypertension, diabetes, glomerulonephritis, or tubulointerstitial damage [1,2]. In addition to the deaths caused by the accumulation of abnormal waste and electrolyte concentration in the body due to the loss of kidney function, it also causes complications such as cardiovascular diseases [1,2].

The experimental animal models of CKD, including aristolochic acid (AA)-induced nephropathy (AAN), puromycin aminonucleoside and adriamycin nephropathies, unilateral streptozotocin (STZ), and ureteral obstruction (UUO), have been established previously [1]. The renal failure-inducing agent, AA, is found in the root extracts of some herbs [3,4]. The earliest record of human AAN was reported in the 1990s, following the use of some weight loss pills containing AA, particularly subtype I of AA (AAI) [3,4]. In addition, AA could be obtained from herbs, food, or some plant extracts [3,4]. The major characteristics of extensive tubulointerstitial fibrosis and the scar formation accompanied by renal tubular atrophy have been found in mouse and human AAN [3,4,5,6,7,8,9]. Recent studies have revealed that AAI causes severe a reduction in the capillaries around the renal tubules, leading to hypoxia and the death of the renal tubular epithelial cells [5,6,7,8,9,10,11]. Furthermore, AAI increased the expression of fibrotic cytokine transforming growth factor 1 (TGF-1) and connective tissue growth factor (CTGF), which enhance fibrosis and scar formation [5,6,11]. According to previous studies, the symptoms of AAN mice are very similar to those in patients with CKD [1,2,3,4].

Metallothioneins (MTs) are cysteine-rich, small intracellular proteins (6–7 kDa) that are capable of binding to heavy metal ions (Zn, Cu, Cd, Hg) [12,13,14]. The four isoforms of MT, including MT-I, MT-II, MT-III, and MT-IV, are encoded on chromosome 16 [15]. MT-I and II appear in several normal mammalian tissues, particularly the liver and the kidney, as well as in tumors. MT-III expression is mainly expressed in brain neurons, while MT-IV is expressed in the epithelial cells of skin and the upper gastrointestinal tract [13,15]. MT-I/II plays a crucial role in metal homeostasis, metal-regulated transcription activity, and detoxification [16,17,18]. They are stimulated by exposure to heavy metals, inflammatory cytokines, reactive oxygen species (ROS), and toxins [12,13,14,17,19,20,21]. Some reports indicated that MT-I could be induced by ischemia or hypoxia and stabilized HIF-1α against hypoxic damage in the kidney [16,22,23,24]. A decreased MT-I expression has been demonstrated in hepatocellular carcinoma (HCC), and the overexpression of MT-I is reported to suppress tumor growth and HCC progression significantly [25]. These results suggest that MT proteins play a crucial protective role in normal tissues and organs.

Although MT expression has been reported in kidney injury [16,22,23,24], whether MT is associated with the stages of CKD and its role in the malignant process of CKD need to be investigated further. In this study, we found that MT-I/II was localized in the renal proximal tubule (PT) and the amount of MT-I/II protein was significantly decreased in the kidneys of CKD patients and chronic AAN mice. Furthermore, we identified a compound, ammonium pyrrolidinedithiocarbamate (PDTC), that significantly restored the AAI-induced MT-I/II reduction in HK2 cells and attenuated the tubulointerstitial fibrosis and nephropathy damage in an AAN mouse model. Thus, we show that reduced MT-I/II expression is associated with advanced CKD, and targeting MT-I/II is a potential therapeutic approach to attenuate the progression to end-stage renal disease (ESRD).

## 2. Results

### 2.1. Expression of MT-I/II in Renal Tissues of Human CKD and Its Clinical Characteristics

A total of 120 patients were retrospectively enrolled in this study with a median age of 65.0 ± 15.1 years old and slight male predominance (62/120, 51.2%) (Table 1). The prevalent renal disease included renal cell carcinoma (RCC), followed by urothelial carcinoma (UC). About 33% (40/120) of the patients had stage 4–5 CKD, and grade 4 histological changes were noted in 29.8% (36/120) of the cases.

**Figure 1:**
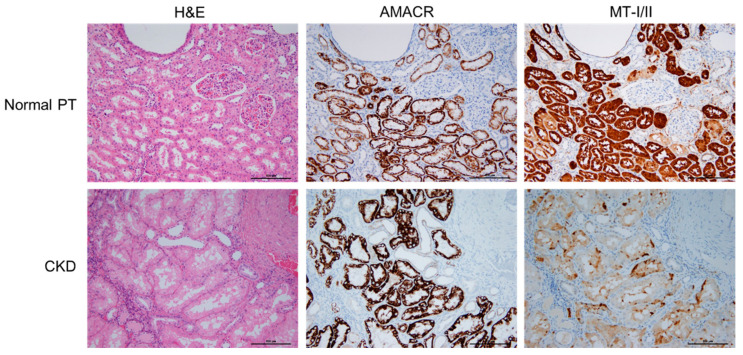
MT-I/II located within proximal tubule (PT) in normal and CKD renal parenchyma. The histopathological images of human kidney slides stained with hematoxylin and eosin (H&E) (Left panel). The location and morphology of PT displayed with alpha-methyl CoA racemase (AMACR) using immunohistochemistry (Middle Panel). Histological images revealed the location and changes of MT-I/II by their specific antibody (Right panel). Bar = 500 μm.

Kidney tissue damage, loss of integrity, and increase in nonfunctional interstitials were observed in normal and CKD kidney tissues using hematoxylin-eosin (H&E) staining (Figure 1, left panel). AMACR marks the position of the proximal tubule (PT). This revealed that both the number and the diameter of functional proximal tubules (PTs) were decreased in the CKD patients (Figure 1, middle panel). The distributions of MT-I/II in the kidney tissue were labeled with specific antibodies and highly correlated with the position of the PT region (Figure 1, right panel). Furthermore, the expression of MT-I/II was significantly reduced in the kidney tissues of the patients with CKD (Figure 1, right panel).

The expression of MT-I/II correlated with underlying renal diseases, while ESRD was significantly lower than others (ESRD vs. others, Q score difference −2.455 ± 0.570, *p* < 0.001) (Table 2). The expression of MT-I/II was also decreased in the advanced functional grades (grade III vs. others, Q score difference −2.062 ± 0.218, *p* < 0.001) and histological grades (grade 4 vs. others, Q score difference −1.909 ± 0.227, *p* < 0.001). Multivariate logistic regression analysis further revealed that the advanced functional grade and the histological grade were independent predictive factors for MT-I/II expression in non-tumor renal tissues (Table 3). 

### 2.2. Expression of MT-I/II in AAI-Induced Nephropathy (AAN) Mouse Model

**Figure 2:**
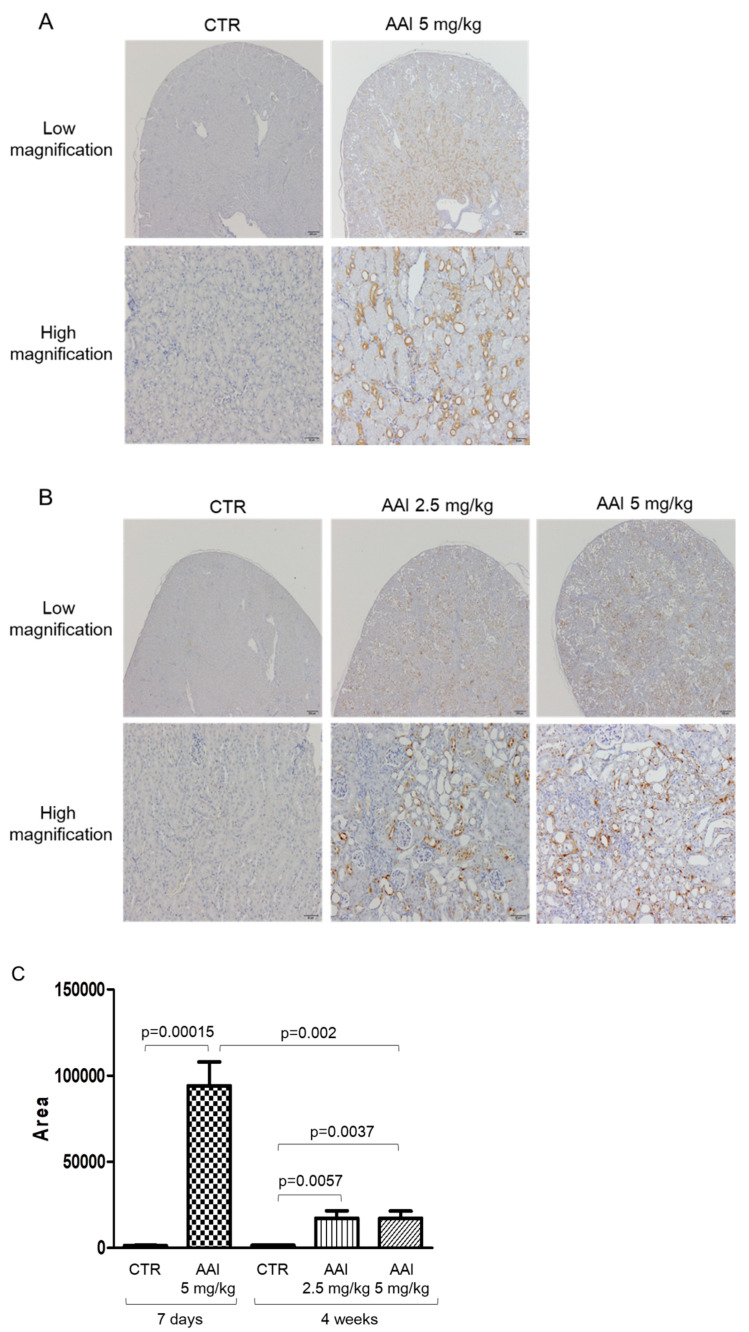
MT-I/II levels in kidney of aristolochic acid (AAI)-induced nephropathy (AAN) mice. (**A**) Histological images of MT-I/II stained using immunohistochemistry in mice without (CTR) or with AAI (5 mg/kg bw) injection for 7 days. (**B**) Histological images of MT-I/II in mice without (CTR) or with AAI injection (2.5 and 5 mg/kg) for 4 weeks. Bar = 200 μm in low magnification image; Bar = 50 μm in high magnification image. (**C**) The statistic figure showing the raw values of distribution area times stain intensity of MT-I/II in (**A**) and (**B**). *n* ≥ 6 in each group.

To elucidate the role of MT-I/II in chronic renal injury, we established a mouse model of kidney damage induced by AAI. Eight to 10-week-old C57BL/6 mice were intraperitoneally injected with AAI for 7 days or 28 days, and MT-I/II expression was examined using immunohistochemistry. The MT-I/II expression was abundantly increased by AAI treatment with an acute induction (7 days) in the renal PT group compared with the control group (representative images in Figure 2A). For a longer period of treatment (28 days), although the expression level at PT was higher than that without AAI treatment (2.5 or 5 mg/kg AAI vs. control group) (Representative image in Figure 2B), the MT-I/II expression was significantly reduced as compared with short-term (7 days) AAI administration (representative images in Figure 2B and statistical analysis in Figure 2C). 

**Figure 3:**
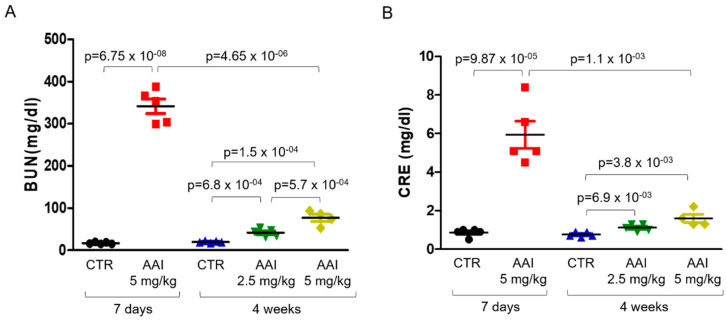
The amounts of serum BUN and CRE in AAN mice. Serum concentrations of (**A**) BUN and (**B**) CRE in mice without (CTR) or with AAI (2.5 and 5 mg/kg) injection detected using ELISA analysis at 7 days and 4 weeks as indicated. *n* ≥ 6 in each group. *P* values of the t-test were denoted as indications.

We then examined the levels of blood urea nitrogen (BUN) (Figure 3A) and creatinine (CRE) (Figure 3B) in the AAN mice sera. The serum BUN and CRE levels were significantly increased in the AAN mice (Figure 3A,B) as compared with the normal group. Similarly, the levels of BUN and CRE were substantially increased in the acute renal injured mice (7 days), but significantly declined in the long-term (4 weeks) AAN-treated mice (Figure 3A,B). Additionally, the body weight of the mice injected with long-term (5 mg/kg) AAI was dramatically decreased as compared with the 2.5 mg/kg AAI-treated mice and the control group (Supplementary Figure S1).

### 2.3. PDTC Restored AAI-Reduced MT-I/II Expression in HK2 Cells

**Figure 4:**
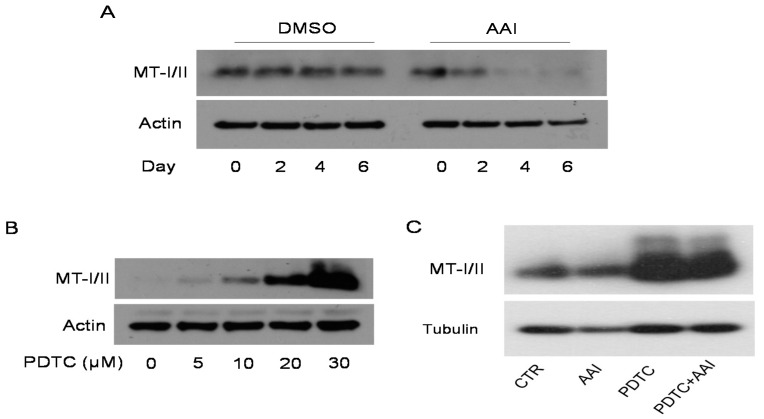
PDTC restored AAI-reduced MT-I/II expression in the HK2 cells. Representative images of (**A**) Expression of MT-I/II treated with 25 µM AAI (DMSO as control) for 0–6 days in HK2 cells. (**B**) Expression of MT-I/II treated with 0–30 µM of PDTC (DMSO as control) for 2 days in HK2 cells. (**C**) MT-I/II expression treated with 25 µM AAI or/and 30 µM PDTC for 2 days in HK2 cells. MT-I/II levels as determined using Western blot analysis. Actin or tubulin was used as the internal loading control.

To elucidate whether AAI affects MT-I/II expression in cultured cells, HK2 cells (kidney PT cell line) were treated with or without 25 µM AAI for 2, 4, and 6 days. The expression of MT-I/II was determined using Western blot analysis. The expression of MT-I/II gradually decreased in a time-dependent manner and was barely detected on days four and six (Figure 4A). As reported by a previous study that PDTC induced MT-I expression through an NFκB-independent mechanism in HCC [25], we examined whether PDTC induced MT-I/II expression in HK2 cells. Our results confirmed that PDTC induced MT-I/II expression in a dose-dependent manner (Figure 4B). To further investigate the effect of PDTC on AAI-reduced MT-I/II expression, the HK2 cells were treated with 25 µM AAI and/or 30 µM PDTC for 2 days. PDTC significantly induced the MT-I/II expression in AAI-treated HK2 cells (Figure 4C).

### 2.4. PDTC Attenuated Kidney Injury in AAN Mice

**Figure 5:**
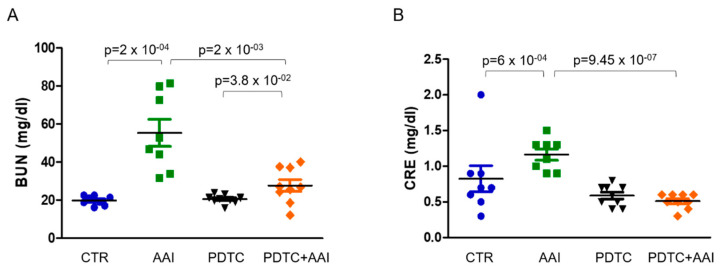
Level of serum BUN and CRE in PDTC treated AAN mice. Serum concentrations of (**A**) BUN and (**B**) CRE in AAN mice injected with 2.5 mg/kg AAI or/and 10 mg/kg PDTC for 4 weeks as determined using ELISA analysis. *n* ≥ 6 in each group. *P* values of the t-test were denoted as indications.

To investigate whether PDTC attenuates AAI-induced renal damage, AAI and/or PDTC was injected into chronic AAN mice. PDTC (10 mg/kg) was injected into 8–10-week-old mice with or without 2.5 mg/kg AAI. The experimental mice were sacrificed on day 28. Both serum BUN and CRE were increased in the AAN mice compared to those without AAI injection (Figure 5A,B). However, the elevated BUN and CRE induced by AAI were attenuated by PDTC treatment (Figure 5A,B). In addition, the amount of renal MTI/II protein in AAI/PDTC treated mice was analyzed using Western blot analysis. PDTC slightly induced MT-1/II expression when compared with AAI treatment, but there is no significant difference between the other groups (Supplementary Figure S2).

**Figure 6:**
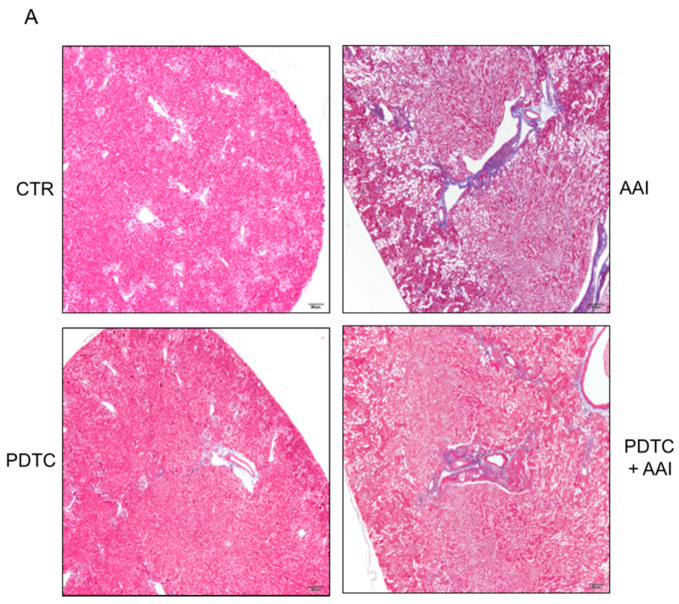

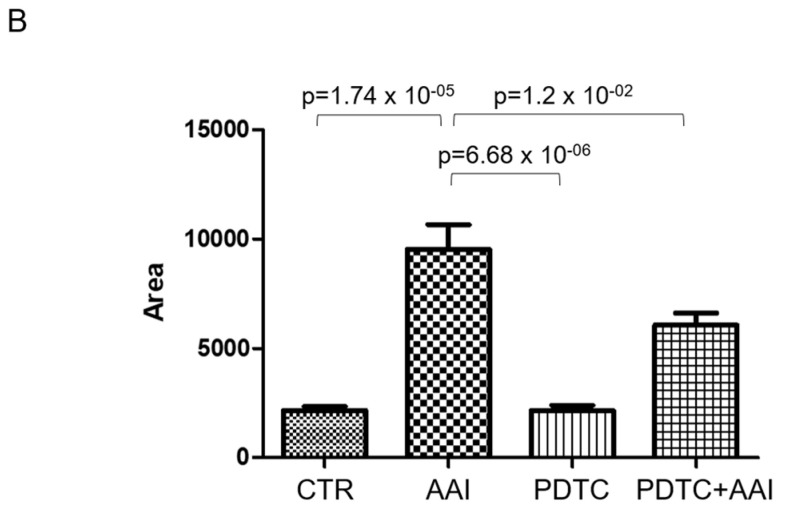
Effect of PDTC for kidney injury in AAN mice. (**A**) The histological analysis of renal fibrosis in AAN (2.5 mg/kg) mice injected with/without PDTC (10 mg/kg) for 4 weeks examined using Masson’s Trichrome stain. Bar = 200 μm. (**B**) The statistical analysis (the raw values of distribution area times stain intensity) of renal fibrosis was performed using image J analysis. *n* ≥ 6 in each group. *P* values of the t-test were denoted as indications.

Further, renal fibrosis was determined in AAN mice injected with or without PDTC, using Masson’s trichrome staining. Renal fibrosis was significantly increased in the AAN mice compared with the control group (Figure 6A,B). Intriguingly, a PDTC injection significantly attenuated the renal fibrosis caused by AAI treatment (Figure 6A,B).

## 3. Discussion

MT-1/II are cysteine-rich, low molecular weight proteins that are capable of directly binding heavy metal ions and reactive oxygen species. Thus, MT-1/II are major protective effectors for scavenging metal toxicity and oxidative stresses. A previous study indicated that MT proteins and glutathione are decreased, and MT expression is relevant to type 2 diabetes mellitus [26]. A significant reduction in MT content has been reported in the peritoneal biopsies of ESRD patients [19]. In this study, we found that MT-I/II is located in the renal tubules of human kidney tissues. In CKD patients, with the atrophy of the renal tubules, the amount of MT-I/II was greatly reduced. A reduced MT-I/II expression was significantly correlated with advanced renal functional grades and ESRD (Table 2 and Table 3). These results indicate that a reduced renal MT-I/II level is associated with advance CKD.

High MT-I/II expression occurs perinatally in the liver and lower expression is seen in the kidney; however, both decrease to the basal levels in adult mice and rats [27]. Here, we showed that AAI treatment induced large amounts of MT-1/II upon intraperitoneal injection for 7 days. However, after 28 days of AAI treatment, the amount of MT-I/II was reduced in the renal tubules of AAN mice. This phenomenon is similar to that reported by a previous study, in which MT-I/II proteins in mouse kidneys were induced by hypoxia for 3 days but reduced on day seven [22]. Additionally, earlier studies have demonstrated that MT-I/II proteins are protective against ischemic renal failure and gentamicin-induced nephrotoxicity in mice and rats [24,28]. This suggests that MT-I/II may play a role in protecting the kidney against stress, heavy metals and/or chemical-induced renal injury. 

Our results demonstrate that the acute phase of MT-I/II was induced by AAI and might be associated with the AAI-related cell protection mechanism under stress. This mechanism may occur due to the inhibition of cell apoptosis and the removal of ROS. Furthermore, long-term AAI treatment induced renal tubule atrophy in the AAN mice, and MT-I/II expression in the renal tubules was significantly decreased. Reduced MT-I/II levels may lead to the impairment renal cell and tissue protective mechanisms. Although the values of serum BUN and CRE in the AAN mice were largely increased in the acute phase, they were ameliorated upon long-term AAI treatment. These results may be caused by physiological compensation, and this phenomenon has also been reported in a previous study [24].

An earlier study indicated that MT-I is expressed in the normal liver, but its expression is reduced in HCC (25). The reduction in MT-I in the liver promotes cancer cell proliferation, tumor growth, and progression. Treatment with PDTC abundantly induced MT-I expression through regulating epigenetic modulation but not an NFκB-dependent pathway in HCC, thereby reducing cancer cell proliferation and tumor growth in a xenograft mouse model [25]. In this study, we demonstrated that AAI reduced MT-I/II expression, whereas PDTC greatly induced MT-I/II but had no effect on DNMT1 expression in HK2 cells (Figure 4 and Supplementary Figure S3). We hypothesize that PDTC may have a protective effect on AAI-induced kidney injury and renal function loss. Our results indicate that PDTC injection significantly improved AAI-induced renal fibrosis and reduced BUN and CRE in the AAN mice (Figure 5 and Figure 6). Instead of inducing MT-I/II, PDTC has been extensively used for NFκB inhibition, metal chelation [29], and anti-oxidative stress [30,31,32]. The molecular mechanism by which PDTC induces MT-1/II expression and attenuates renal injury in AAN warrants further investigation.

In conclusion, our results reveal that MT-I/II may play a crucial role in protection against renal injury/failure. The reduced MT-I/II levels significantly correlate with advanced functional grades of CKD and ESRD in human patients. The administration of PDTC in HK2 cells induced MT-I/II expression and ameliorated AAI-induced renal fibrosis and kidney function loss in the AAN mice. Therefore, developing approaches to maintain renal MT-I/II levels may be a potential therapeutic strategy for CKD. 

## 4. Materials and Methods

### 4.1. Patients and Specimens

A total of 120 patients who underwent nephrectomy were enrolled in this study. Renal tissue specimens were obtained from the tissue bank of the Taichung Veterans General Hospital, Taichung, Taiwan. The current study, along with the policy of waiving informed consents, was approved by the Institutional Review Board of Taichung Veterans General Hospital (No. CE16146B). The clinicopathological characteristics of the patients are presented in Table 1. The median age of patients was 65.0 ± 15.1 years, including 62 males and 58 females. The pathological diagnoses included 53 renal cell carcinomas (RCCs), 42 urothelial carcinomas (UCs), 6 angiomyolipomas (AMLs), 11 ESRDs, one oncocytoma, one metastatic HCC, one metanephric adenoma, one pyelonephritis, one leiomyoma, one schwannoma, one spindle cell sarcoma, and one urothelial neoplasm of low malignant potential (UNLMP). The mean estimated glomerular filtration rate (eGFR) (excluding dialysis) was 66.8 ± 27.6 mL/min/1.73 m^2^. The functional grade was stratified into three levels, according to the nearest eGFR, prior to the time of surgery. Grade 1 (normal functions) was referred for those patients with eGFR ≥ 60 mL/min/1.73 m^2^, grade 2 (impaired function) for eGFR ≥ 15 mL/min/1.73 m^2^ but < 60 mL/min/1.73 m^2^, and grade 3 (ESRD) for those with eGFR < 15 mL/min/1.73 m^2^ or on dialysis.

Histological grade was stratified into five grades based on the histological degree of renal injury on non-neoplastic renal tissue. The injured renal parenchyma may show tubular necrosis, tubular atrophy, decreased tubular density, glomerular sclerosis, fibrosis, and inflammatory cell infiltration. The degree of renal injury was examined and graded on one representative tissue section using hematoxylin and eosin (H&E) staining. Renal injury < 1% was defined as grade 1, renal injury ≥ 1% but < 50% as grade 2, renal injury ≥ 50% but < 99% as grade 3, and renal injury ≥ 99% as grade 4. Grade 4 renal injury was subclassified into grades 4a, defined as the presence of a recognizable proximal tubule on H&E, and grade 4b, defined as the absence of a recognizable PT on H&E. 

### 4.2. Immunohistochemical Stain of Human Kidney Tissues

Immunohistochemical staining was performed on formalin-fixed paraffin-embedded tissue sections using an automatic immunostaining device and OptiView detection kit (Ventana XT Medical System, Tucson, AZ, USA). MT-I/II expression in tissue sections was analyzed using immunostaining with an anti-MT-I/II antibody (clone: E9; 1:300 dilution; Invitrogen). The target for evaluating MT-I/II expression was the proximal tubule. The immunostaining of MT-I/II was scored semi-quantitatively using the Quick score (Q-score) method [33,34,35,36,37,38,39], based on the intensity and proportion of the positive cells of the stain. The intensity of staining was scored as 0 (negative), 1 (weak), 2 (moderate), or 3 (strong). The percentage score was defined as the proportion of the number of MT-I/II-stained proximal tubules to the number of proximal tubules in the normal kidney, and it was scored as 0 (0%), 1 (1–25%), 2 (26–50%), 3 (51–75%), and 4 (76–100%). The sum of the intensity score and percentage score yields a Q-score, which ranges from to 0–7. 

### 4.3. Cell Culture and Reagents

The HK2 cell line (American Type Culture Collection, ATCC-CRL2190) was cultured in Dulbecco’s modified Eagle’s medium (DMEM) supplemented with 10% fetal bovine serum (FBS), 100 U/mL penicillin, and 100 mg/mL streptomycin at 37 °C in a humidified 5% CO_2_ atmosphere. Ammonium pyrrolidine dithiocarbamate (PDTC) and dimethyl sulfoxide (DMSO) were purchased from Sigma-Aldrich (St. Louis, MO, USA). AAI (Subtype I of AA) was purchased from Cayman Chemical Company (Ann Arbor, MI, USA). Masson’s trichrome stain kit was purchased from ScyTek Laboratories (Logan, UT, USA). The metallothionein (MT) antibody was purchased from Invitrogen Corporation (Camarillo, CA, USA). DMEM, FBS, MEM non-essential amino acids (NEAA), penicillin, and streptomycin for cell culture were obtained from Invitrogen-Gibco (Grand Island, NY, USA).

### 4.4. Western Blot Analysis

The cells were lysed in ice-cold RIPA buffer (Millipore, Temecula, CA, USA) containing a protease inhibitor cocktail (Roche, IN, USA). Cell lysates were centrifuged at 15,000 rpm for 20 min at 4 °C, and total protein concentrations were determined using a Bio-Rad protein assay kit (Bio-Rad Laboratories). Equal amounts of total protein from each sample were run through a gradient SDS-PAGE gel, followed by immunoblotting onto PVDF membranes. The membranes were blocked and probed with primary antibodies against actin (Sigma-Aldrich), tubulin (Sigma-Aldrich), and MT-I/II (Invitrogen). The membranes were immersed in 0.1% PBST containing horseradish peroxidase-conjugated secondary antibodies, and the protein levels were determined using enhanced chemiluminescence reagents.

### 4.5. Experimental Animals

All experimental procedures were performed in accordance with the guidelines and regulations approved by the Institutional Animal Care and Use Committee of the National Health Research Institutes. Eight to 10-week-old C57BL/6 mice were purchased from the National Laboratory Animal Center and housed in microisolator cages in a specific pathogen-free facility at the National Health Research Institutes. AAN was induced in C57BL/6J mice by intraperitoneal injection of AAI. Acute kidney injury was induced by administration of AAI (5 mg/kg) every day for 7 days [40], and chronic renal injury was carried out by the administration of AAI (2.5 mg or 5 mg/kg) once every 2 days for 4 weeks [1,2]. The control animals received an intraperitoneal injection of vehicle control (DMSO) diluted in an equal volume of phosphate-buffered saline (PBS) solution. PDTC (10 mg/kg) was injected intraperitoneally with/without AAI at the same time once every 2 days for 4 weeks. All the procedures were carried out under license, according to the regulations laid down by Her Majesty’s Government, United Kingdom (Animals Scientific Procedures Act, 1986).

### 4.6. Immunohistochemical Analysis and Measurement of Renal Fibrosis

For immunohistochemical analysis, kidney tissues from the sacrificed mice were harvested and fixed in 10% formaldehyde. Paraffin cross-sections with a thickness of 5 μm were stained with antibodies against MT-I/II (Invitrogen), followed by N-Histofine^®^ MOUSESTAIN KIT (414321F; Nichirei Biosciences Inc.; Tokyo, Japan). To measure renal fibrosis, the paraffin-embedded sections were stained using Masson’s Trichrome stain kit (ScyTek Laboratories Inc., Logan, USA). Quantification of kidney immunohistochemical assay and fibrosis on Masson’s trichrome-stained sections were performed using ImageJ analysis. The raw pixel values of the selected areas were estimated using the ImageJ software. These values indicated the distribution area times the stain intensity of the images.

### 4.7. Statistical Analysis

Kruskal–Wallis one-way ANOVA test and post hoc Dunnett’s test were utilized to identify the correlation of MT-I/II expression with the clinicopathological parameters. Multivariate logistic regression was used to analyze the factors affecting MT-I/II expression in non-tumor renal tissues. For the results of the AAN mouse model, data were analyzed using Student’s *t*-test between different groups. All statistical analyses were performed using SPSS Statistics version 23 (IBM) for Windows. Statistical significance was set at *p* < 0.05.

## Figures and Tables

**Table 1:** Demographics of the patients with available renal pathological specimens. AML, angiomyolipoma; CKD, chronic kidney disease; ESRD, end-stage renal disease; GFR, glomerular filtration rate; RCC, renal cell carcinoma; SD, standard deviation; UC, urothelial carcinoma.

Characters	Number (%)
Total	120 (100%)
Age (y/o)	
Median ± SD	65.0 ± 15.1
20–29	1 (0.8%)
30–39	12 (9.9%)
40–49	11 (9.1%)
50–59	18 (14.9%)
60–69	32 (26.4%)
70–79	30 (24.8%)
≥80	16 (13.2%)
Gender	
Male	62 (51.2%)
Female	58 (47.9%)
Diagnosis	
RCC	53 (43.8%)
UC	42 (34.7%)
AML	6 (5.0%)
ESRD	11 (9.1%)
Other	8 (6.6%)
CKD stage	
1	10 (8.3%)
2	41 (33.9%)
3	29 (24.0%)
4	5 (4.1%)
5	35 (28.9%)
Functional grade	
I	51 (42.1%)
II	34 (28.1%)
III	35 (28.9%)
eGFR (excluding dialysis)	
Mean ± SD	66.8 ± 27.6
Histology Grade	
1	37 (30.6%)
2	30 (24.8%)
3	17 (14.0%)
4A	14 (11.6%)
4B	22 (18.2%)

**Table 2:** Correlation of non-tumor part MT-I/II expression with clinico-pathological parameters. AML, angiomyolipoma; CKD, chronic kidney disease; ESRD, end-stage renal disease; RCC, renal cell carcinoma; SD, standard deviation; UC, urothelial carcinoma.

Characters	Non-Tumor Part Q-Score	*p*-Value
	(Mean ± SD)	
Age		
<65 y/o	5.16 ± 1.14	0.184
≥65 y/o	4.81 ± 1.45	
Gender		
Male	4.82 ± 1.41	0.230
Female	5.14 ± 1.46	
Diagnosis		
RCC	5.43 ± 1.08	<0.001
UC	4.36 ± 1.43	
AML	6.50 ± 1.23	
ESRD	3.55 ± 0.69	
Others	6.00 ± 1.51	
Functional grade		
I	5.86 ± 1.18	<0.001
II	5.15 ± 1.13	
III	3.51 ± 0.72	
Histology Grade		
1	6.76 ± 0.44	<0.001
2	4.77 ± 0.79	
3	4.29 ± 0.92	
4A	3.86 ± 0.77	
4B	3.50 ± 0.74	

**Table 3:** Multivariate analysis of clinico-pathological parameters on non-tumor part MT-1/II expression. AML, angiomyolipoma; CKD, chronic kidney disease; ESRD, end-stage renal disease; RCC, renal cell carcinoma; UC, urothelial carcinoma.

Parameters	Estimated	95% Confident Intervals	*p*-Value
Age			
<65 y/o	1		
≥65 y/o	0.989	0.757–1.292	0.934
Gender			
Male	1		
Female	1.326	1.037–1.697	0.025
Diagnosis			
RCC	1		
UC	0.813	0.578–1.144	0.232
ESRD	0.894	0.534–1.496	0.666
AML + Others	0.892	0.591–1.347	0.583
Histology Grade			
1	1		
2 + 3	0.130	0.097–0.175	<0.001
4A + 4B	0.089	0.287–0.718	<0.001
Functional grade			
I	1		
II	1.113	0.781–1.585	0.550
III	0.454	0.287–0.718	0.001

## Data Availability

Data are available in a publicly accessible repository that does not issue DOIs.

## Supplementary Materials

The following are available online at www.mdpi.com/article/10.3390/toxins13080568/s1, Figure S1: Body weight of AAN mice. Body weight of mice without (CTR) or with AAI (2.5 and 5 mg/kg) injection were detected at indicated days. *n* ≥ 6 in each group, Figure S2: The representative result of renal MT-I/II protein in AAN/PDTC treated mice determined by Western blot analysis, Figure S3: The representative result of DNMT1 expression AAI-treated HK2 cells determined by Western Blot analysis.

## References

[1] Huang L., Scarpellini A., Funck M., Verderio E.A., Johnson T.S. (2013). Development of a Chronic Kidney Disease Model in C57BL/6 Mice with Relevance to Human Pathology. Nephron Extra.

[2] Rabe M., Schaefer F. (2016). Non-Transgenic Mouse Models of Kidney Disease. Nephron.

[3] Debelle F.D., Vanherweghem J.-L., Nortier J.L. (2008). Aristolochic acid nephropathy: A worldwide problem. Kidney Int..

[4] Yang B., Xie Y., Guo M., Rosner M.H., Yang H., Ronco C. (2018). Nephrotoxicity and Chinese herbal medicine. Clin. J. Am. Soc. Nephrol..

[5] Chang J.-F., Hsieh C.-Y., Lu K.-C., Chen Y.-W., Liang S.-S., Lin C.-C., Hung C.-F., Liou J.-C., Wu M.-S. (2020). Therapeutic Targeting of Aristolochic Acid Induced Uremic Toxin Retention, SMAD 2/3 and JNK/ERK Pathways in Tubulointerstitial Fibrosis: Nephroprotective Role of Propolis in Chronic Kidney Disease. Toxins.

[6] Kim J.-Y., Leem J., Jeon E.J. (2019). Protective Effects of Melatonin Against Aristolochic Acid-Induced Nephropathy in Mice. Bio-Molecules.

[7] Nortier J.L., Deschodt-Lanckman M.M., Simon S., Thielemans N.O., de Prez E.G., Depierreux M.F., Tielemans C.L., Richard C., Lauwerys R.R., Bernard A.M. (1997). Proximal tubular injury in Chinese herbs nephropathy: Monitoring by neutral endopeptidase enzymuria. Kidney Int..

[8] Zhao H., Jiang N., Han Y., Yang M., Gao P., Xiong X., Xiong S., Zeng L., Xiao Y., Wei L. (2020). Aristolochic acid induces renal fibrosis by arresting proximal tubular cells in G2/M phase mediated by HIF-1α. FASEB J..

[9] Sun D., Feng J., Dai C., Sun L., Jin T., Ma J., Wang L. (2006). Role of Peritubular Capillary Loss and Hypoxia in Progressive Tubulointerstitial Fibrosis in a Rat Model of Aristolochic Acid Nephropathy. Am. J. Nephrol..

[10] Guo Y., Hu M., Ma J., Chinnathambi A., Alharbi S.A., Shair O.H.M., Ge P. (2021). Protective effect of panaxydol against repeated administration of aristolochic acid on renal function and lipid peroxidation products via activating Keap1-Nrf2/ARE pathway in rat kidney. J. Biochem. Mol. Toxicol..

[11] Yang L., Li X., Wang H. (2006). Possible mechanisms explaining the tendency towards interstitial fibrosis in aristolochic acid-induced acute tubular necrosis. Nephrol. Dial. Transplant..

[12] Chiaverini N., De Ley M. (2010). Protective effect of metallothionein on oxidative stress-induced DNA damage. Free. Radic. Res..

[13] Chmielewska M., Symonowicz K., Pula B., Owczarek T., Podhorska-Okolow M., Ugorski M., Dziegiel P. (2015). Expression of metallothioneins I and II in kidney of doxorubicin-treated rats. Exp. Toxicol. Pathol..

[14] Haq F. (2003). Signaling events for metallothionein induction. Mutat. Res. Mol. Mech. Mutagen..

[15] Thirumoorthy N., Sunder A.S., Kumar K.M., Kumar M.S., Ganesh G., Chatterjee M. (2011). A Review of Metallothionein Isoforms and their Role in Pathophysiology. World J. Surg. Oncol..

[16] Bauer R. (2009). Metallothionein: A new soldier in the fight against chronic renal hypoxia?. Kidney Int..

[17] Ling X.-B., Wei H.-W., Wang J., Kong Y.-Q., Wu Y.-Y., Guo J.-L., Li T.-F., Li J.-K. (2016). Mammalian Metallothionein-2A and Oxidative Stress. Int. J. Mol. Sci..

[18] Vašák M. (2005). Advances in metallothionein structure and functions. J. Trace Elements Med. Biol..

[19] Alscher D.M., Biegger D., Mettang T., Dunst R., Wolken D., Kuhlmann U., Fritz P. (2001). Peritoneal metallothionein content in patients with end-stage renal disease on or not on peritoneal dialysis. Perit. Dial. Int..

[20] Subramanian Vignesh K., Deepe G.S. (2017). Metallothioneins: Emerging Modulators in Immunity and Infection. Int. J. Mol. Sci..

[21] Takahashi S. (2012). Positive and negative regulators of the metallothionein gene (Review). Mol. Med. Rep..

[22] Kojima I., Tanaka T., Inagi R., Nishi H., Aburatani H., Kato H., Miyata T., Fujita T., Nangaku M. (2009). Metallothionein is upregulated by hypoxia and stabilizes hypoxia-inducible factor in the kidney. Kidney Int..

[23] Takahashi T., Itano Y., Noji S., Matsumoto K., Taga N., Mizukawa S., Toda N., Matsumi M., Morita K., Hirakawa M. (2001). Induction of renal metallothionein in rats with ischemic renal failure. Res. Commun. Mol. Pathol. Pharmacol..

[24] Wu H., Zhou S., Kong L., Chen J., Feng W., Cai J., Miao L., Tan Y. (2015). Metallothionein deletion exacerbates intermittent hypoxia-induced renal injury in mice. Toxicol. Lett..

[25] Wu Y.-J., Ko B.-S., Liang S.-M., Lu Y.-J., Jan Y.-J., Jiang S.-S., Shyue S.-K., Chen L., Liou J.-Y. (2019). ZNF479 downregulates metallothionein-1 expression by regulating ASH2L and DNMT1 in hepatocellular carcinoma. Cell Death Dis..

[26] Raudenska M., Dvorakova V., Pacal L., Chalasova K., Kratochvilova M., Gumulec J., Ruttkay-Nedecky B., Zitka O., Kankova K., Adam V. (2017). Levels of heavy metals and their binding protein metallothionein in type 2 diabetics with kidney disease. J. Biochem. Mol. Toxicol..

[27] Ljubojević M., Orct T., Micek V., Karaica D., Jurasović J., Breljak D., Madunić I.V., Rašić D., Jovanović I.N., Peraica M. (2019). Sex-dependent expression of metallothioneins MT1 and MT2 and concentrations of trace elements in rat liver and kidney tissues: Effect of gonadectomy. J. Trace Elements Med. Biol..

[28] Yang C.-L., Du X.-H., Zou W.-Z., Chen W. (1991). Protective Effect of Zinc-Induced Metallothionein Synthesis on Gentamicin Nephrotoxicity in Rats. Ren. Fail..

[29] Moon S.-K., Jung S.-Y., Choi Y.-H., Lee Y.-C., Patterson C., Kim C.-H. (2003). PDTC, metal chelating compound, induces G1 phase cell cycle arrest in vascular smooth muscle cells through inducing p21Cip1 expression: Involvement of p38 mitogen activated protein kinase. J. Cell. Physiol..

[30] Haddad J.J.E., Olver R.E., Land S. (2000). Antioxidant/Pro-oxidant Equilibrium Regulates HIF-1α and NF-κB Redox Sensitivity. J. Biol. Chem..

[31] Schmitt A., Brändle A.-L., Herzog P., Röchner F., Fragasso A., Munz B. (2020). Effects of the anti-oxidant PDTC in combination with a single bout of treadmill running on murine skeletal muscle. Redox Rep..

[32] Zhu B.-Z., Carr A.C., Frei B. (2002). Pyrrolidine dithiocarbamate is a potent antioxidant against hypochlorous acid-induced protein damage. FEBS Lett..

[33] Barnes D.M., Harris W.H., Smith P., Millis R.R., Rubens R.D. (1996). Immunohistochemical determination of oestrogen receptor: Comparison of different methods of assessment of staining and correlation with clinical outcome of breast cancer patients. Br. J. Cancer.

[34] Chang G.-C., Liu K.-J., Hsieh C.-L., Hu T.-S., Charoenfuprasert S., Liu H.-K., Luh K.-T., Hsu L.-H., Wu C.-W., Ting C.-C. (2006). Identification of α-Enolase as an Autoantigen in Lung Cancer: Its Overexpression Is Associated with Clinical Outcomes. Clin. Cancer Res..

[35] Jan Y.-J., Ko B.-S., Hsu C., Chang T.-C., Chen S.-C., Wang J., Liou J.-Y. (2009). Overexpressed focal adhesion kinase predicts a higher incidence of extrahepatic metastasis and worse survival in hepatocellular carcinoma. Hum. Pathol..

[36] Ko B.-S., Chang T.-C., Hsu C., Chen Y.-C., Shen T.-L., Chen S.-C., Wang J., Wu K.K., Jan Y.-J., Liou J.-Y. (2011). Overexpression of 14-3-3? predicts tumour metastasis and poor survival in hepatocellular carcinoma. Histopathology.

[37] Liu T.-A., Jan Y.-J., Ko B.-S., Chen S.-C., Liang S.-M., Hung Y.-L., Hsu C., Shen T.-L., Lee Y.-M., Chen P.-F. (2011). Increased Expression of 14-3-3β Promotes Tumor Progression and Predicts Extrahepatic Metastasis and Worse Survival in Hepatocellular Carcinoma. Am. J. Pathol..

[38] Liu T.-A., Jan Y.-J., Ko B.-S., Liang S.-M., Chen S.-C., Wang J., Hsu C., Wu Y.-M., Liou J.-Y. (2013). 14-3-3ε Overexpression Contributes to Epithelial-Mesenchymal Transition of Hepatocellular Carcinoma. PLoS ONE.

[39] Lu Y.-J., Jan Y.-J., Ko B.-S., Liang S.-M., Chen L., Wu C.-C., Chin C.-H., Kuo C.-C., Yet S.-F., Liou J.-Y. (2020). Expression of Nik-related kinase in smooth muscle cells attenuates vascular inflammation and intimal hyperplasia. Aging.

[40] Baudoux T.E., Pozdzik A.A., Arlt V.M., De Prez E.G., Antoine M.-H., Quellard N., Goujon J.-M., Nortier J.L. (2012). Probenecid prevents acute tubular necrosis in a mouse model of aristolochic acid nephropathy. Kidney Int..

