# Supplementary material for: Reduced Expression of Metallothionein-I/II in Renal Proximal Tubules Is Associated with Advanced Chronic Kidney Disease
Source: Toxins (Basel). 2021 Aug 15;13(8):568. doi: 10.3390/toxins13080568 (PMC8402552; doi:10.3390/toxins13080568)
Supplement: Supplementary file 1 [file toxins-13-00568-s001.zip › toxins-1228632-supplementary.pdf]

Supplementary Materials: Reduced Expression of Metallothionein-I/II in Renal Proximal Tubules Is Associated with Advanced Chronic Kidney Disease

Yi-Jhu Lu, Ya-Ju Wu, Lujen Chen, Bor-Sheng Ko, Tzu-Ching Chang, Yi-Ju Wu, Shu-Man Liang, Yee-Jee Jan and Jun-Yang Liou

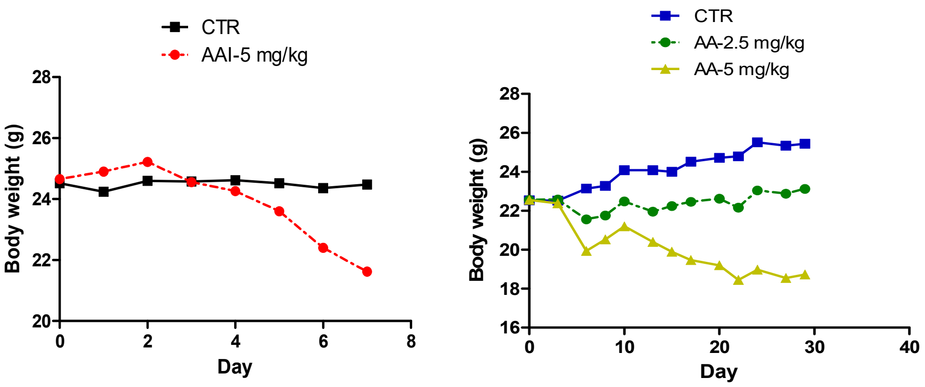

Figure S1. Body weight of AAN mice. Body weight of mice without (CTR) or with AAI (2.5 and 5 mg/kg) injection were detected at indicated days. *n* ≥6 in each group.

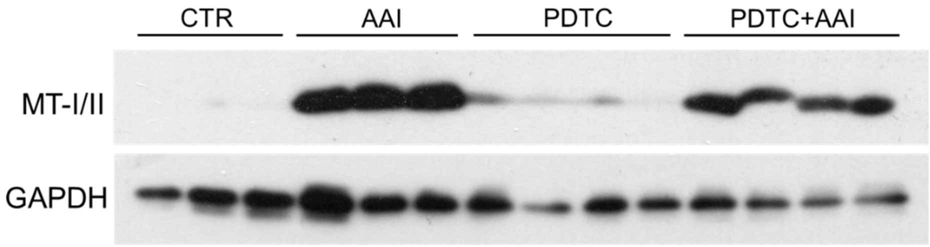

Figure S2. The representative result of renal MT-I/II protein in AAN/PDTC treated mice determined by Western blot analysis.

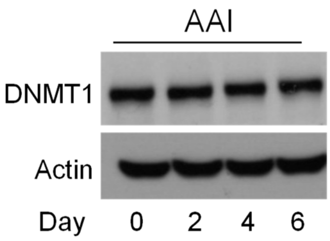

Figure S3. The representative result of DNMT1 expression AAI-treated HK2 cells determined by Western Blot analysis.
